# Estimation of the Frequency and Time of Human Exposure to Arsenic by Single Hair Analysis

**DOI:** 10.3390/ijerph191811429

**Published:** 2022-09-11

**Authors:** Bing Li, Weicong Xu, Ruxin Luo, Shaojie Zhuo, Xueyan Guo, Kuan Cheng, Keming Yun, Dong Ma

**Affiliations:** 1 School of Forensic Medicine, Shanxi Medical University, Taiyuan 030001, China; 2 Jinan University Center of Forensic Science, Jinan University, Guangzhou 510630, China; 3 School of Public Health, Southern Medical University, Guangzhou 510515, China; 4 Shanghai Key Laboratory of Forensic Medicine, Key Laboratory of Forensic Science, Ministry of Justice, Shanghai Forensic Service Platform, Academy of Forensic Science, Shanghai 200063, China

**Keywords:** arsenic, LA-ICP-MS, single hair, hair growth rate, frequency, exposure time

## Abstract

Arsenic (As) and its compounds are widely used in many applications. Long-term exposure to As can cause acute and chronic poisoning. In severe cases, it can lead to adverse effects, such as gene mutation, cell cancer and fetal malformation. The objective of this study was to accurately estimate As exposure frequency and time. Quantitative analysis of As in single hairs obtained from APL (acute promyelocytic leukemia) patients treated with As_2_O_3_ was performed by LA-ICP-MS. An informative As concentration distribution profile of single hair was applied to estimate the As exposure frequency and time. As exposure frequency was estimated according to the number of As concentration peaks. As exposure time was estimated according to the hair growth length in combination with the hair growth rate. The validation results demonstrate that this method was more efficient than the traditional method; compared with the traditional method, which provides estimates in months, our model shortened the As exposure time estimate to the range of a few days, which considerably improved the inference accuracy. Therefore, these results can be used for forensic toxicology studies, environmental exposure monitoring, etc.

## 1. Introduction

Arsenic (As) is a metal element that is widely present in surface, soil, water, the atmosphere, food and organisms in the form of compounds. Extended exposure to As-containing environments can cause As poisoning in human. As poisoning has not only become a popular issue in the field of public health research but a common health problem encountered worldwide. Exposure to As is a global public health concern closely associated with numerous adverse effects [1]. Therefore, it is essential for forensic and environmental scientists to infer when As enters organisms, in addition to other research questions with respect to this topic.

Hair toxicology analysis is of increasing importance in studies related to medicine [2,3], forensics [4], archaeology [5,6] and environmental monitoring [7,8,9]. The analysis of hair has certain advantages compared with blood and urine analyses. Hair can be noninvasively collected with minimal cost and is easily stored and transported to the laboratory for analysis [10]. Additionally, blood and urine are only suitable for monitoring short-term exposure, owing to their rapid pharmacokinetics [11,12], whereas hair can be used to assess historic exposure to elements [13], as trace elements have a special affinity with hair, and hair has a strong recording function, which can reflect environmental exposure levels in the body, as well as long-term intake concentrations in tissues.

Human occipital hair has a relatively stable growth rate; however, the use of occipital hair to assess poisoning time remains a challenging task. In previous studies, hairs were mainly analyzed after segmentation and homogenization of a large amount of hair samples [14,15,16]. However, due to the poor spatial resolution, these studies were unable to provide an accurate exposure time. LA-ICP-MS has been proven a powerful tool for directly sampling hair with both in real time and in situ [17]. This method has high sensitivity, low sample consumption, fast detection speed, low detection limit and a wide linear range and can measure multiple elements at the same time. Therefore, it has been increasingly used in biological sample analysis.

The determination of As in hair can be used as a biomonitoring index of occupational exposure and environmental pollution for retrospective investigation and analysis. In this study, accurate determination of As exposure frequency and time are discussed. To the best of our knowledge, this is the first time study to include a discussion of the accuracy As exposure frequency and time estimation.

## 2. Material and Methods

### 2.1. Instrumentation and Reagents

An Agilent 7500Ce inductively coupled plasma mass spectrometer (Agilent Technologies Inc., Palo Alto, CA, USA) was coupled with a UP 213 New Wave laser ablation system (Cambridge, UK). A standard reference material (NIST 612) was used only to tune the ablation system. The experimental parameters (RF power: 1500 W; carrier gas flow rate: 0.87 L/min) of the LA-ICP-MS measurements were optimized to obtain the maximum analytical ion (M^+^) intensity, the minimum oxide (MO^+^) intensity and double-charged (M^2+^) ions. High-purity deionized water obtained by a Milli-Q laboratory water purification system (Millipore, MA, USA) and acetone (Shanghai Ling Feng Chemical Reagent Co., LTD., Shanghai, China) were used throughout. Detailed information was described in our previous study [17].

### 2.2. Sample Preparation and Sample Analysis

Hair samples were obtained from APL (acute promyelocytic leukemia) patients (No. 1 to 8) who were treated with arsenic trioxide (As_2_O_3_) as a therapeutic drug, never having been occupationally or environmentally exposed to As. Five patients were randomly selected to estimate the growth rate of hair and establish a mathematical model for estimation of As exposure time, and the other three patients were used to verify the accuracy of this mathematical model. Detailed information about the medical profiles of all patients is summarized in Table 1.

We cut 10 hairs from each patient as samples. Hair was cut close to the root, and the sampling area was limited to the occipital region of the head. Hair samples were washed with a 1:200 *v*/*v* dilution of Triton X-100, deionized water and acetone [18] and dried in an oven at 75 ± 5 °C. Hair samples were fixed onto double-sided adhesive tape and analyzed via LA-ICP-MS. The hair was ablated from the root to the tip using single-spot scan mode. The diameter of each ablated spot was 55 μm, and the distance between two adjacent spots was 15 μm.

### 2.3. Calibration Strategy

Quality control hairs were analyzed to verify the accuracy and precision of the developed LA-ICP-MS method for elemental quantification in hair. Satisfactory accuracy (97.0%, 107.4%) was obtained with respect to elemental quantification, and the RSDs (precision) were less than 5.6%.

### 2.4. Data Processing

Simple descriptive statistics were performed in Excel 2019 (Microsoft Corp., Redmond, WA, USA). Analysis of variance (ANOVA) and least significance difference (LSD) tests were carried out to test the variance of the hair growth rates among five patients.

### 2.5. Ethics Statement

The patients and one-victim of arsenic poisoning agreed to participate in this study and were informed that As concentration in their hair samples would be determined. The study was approved by the Ethics Committee of Academy of Forensic Science, Shanghai, China (approval code 2018-01-03). Written informed consent was obtained from all participants. All experimental methods involving human participants were conducted in accordance with the 1975 Declaration of Helsinki.

## 3. Results

### 3.1. Representativeness of Single Hair Analysis

**Figure 1:**
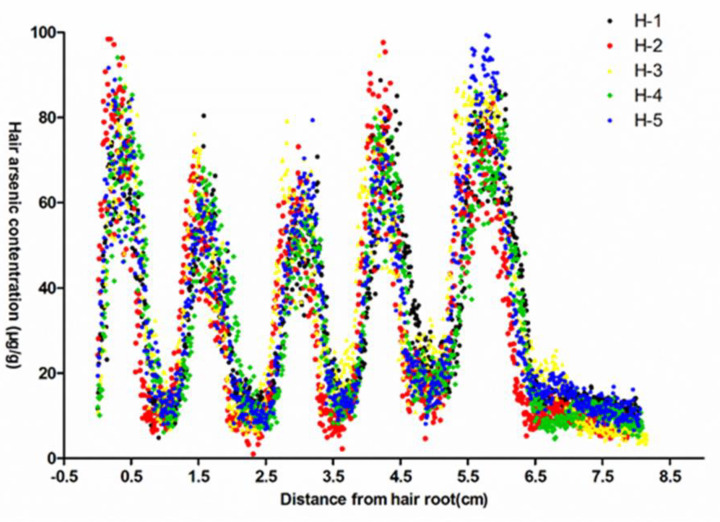
Arsenic concentration profiles of five hairs collected from the occipital region of patient No. 1. H-1 to H-5 represented hair samples 1 to 5, respectively.

The representativeness of single hair analysis was investigated to determine whether different hairs from the same occipital region had the same As concentration distribution profile. Five hairs from the occipital region were collected and ablated according to the same procedure as that described above. The analysis results of the patient No. 1 are shown in Figure 1. Different hairs in the occipital region exhibited the same change trend with respect to As, and the positions of As concentration peaks almost coincided between hairs, indicating that different hairs collected from occipital region had the same As concentration distribution profiles. Similar results were obtained from four others(not shown here). Therefore, the single hair can be used to estimate As exposure frequency and time.

### 3.2. Estimation of As Exposure Frequency

**Figure 2:**
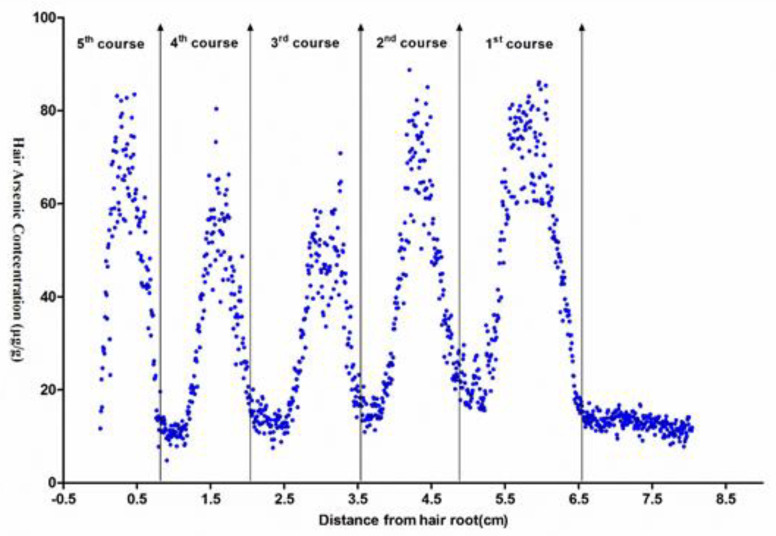
Arsenic exposure frequency for patient No. 1.

The hairs obtained from five patients (patients No. 1 to 5) were ablated from the root to the tip using single-spot scan mode. The As concentration distribution profile along the hair was plotted. The horizontal axis indicates the distance from the hair root and the vertical axis, representing the As concentration along hair. Taking patient No. 1 as an example, as shown in Figure 2, the analysis results demonstrate that the As concentration distribution profile precisely reflects the variations in absorbed As and exposure frequency. There were five As concentration cycles, which is in accordance with the clinical background of patient No. 1, who had received an As_2_O_3_ treatment of five courses during the period of therapy. The same results were observed for all patients (not shown here). Therefore exposure frequency could be inferred based on the numbers of As concentration peaks.

### 3.3. Estimation of Occipital Region Hair Growth Rate

In this study, the hair growth rate was estimated by dividing the hair length by the interval between the days at the beginning of the As_2_O_3_ treatments of two adjacent courses. The ablating procedure was executed in a total of three hairs collected from each patient. The sampling area was limited to the occipital region of the head.

As shown in Table 2, the hair growth rates of the five patients (patient Nos. 1 to 5) ranged from 398 to 439 μm/day, with an average of 412 μm/day and a standard deviation (SD) of the mean of 8 μm/day. ANOVA (analysis of variance) was used to test the variance of the hair growth rates among the five patients, with a *p* value > 0.05 (*p* = 0.426) indicating - no difference in results. The result demonstrate that there were no significant differences in hair growth rates between patients, and the growth rates were consistent between different hairs from the occipital region.

### 3.4. Establishment and Validation of Mathematical Model for Exposure Time Estimation

In this study, As exposure time was estimated according to the hair growth length corresponding to variations in the arsenic concentration, in combination with an estimated hair growth rate of 412 μm/day. A mathematical model was established as follows.

(1) $$\mathrm{Estimated}\;\mathrm{Time}\;(\mathrm{day})\;=\frac{\mathrm{the}\;\mathrm{Hair}\;\mathrm{Growth}\;\mathrm{Length}\;(\mathrm{cm})}{412\;(\mu m/\mathrm{day})}$$

**Figure 3:**
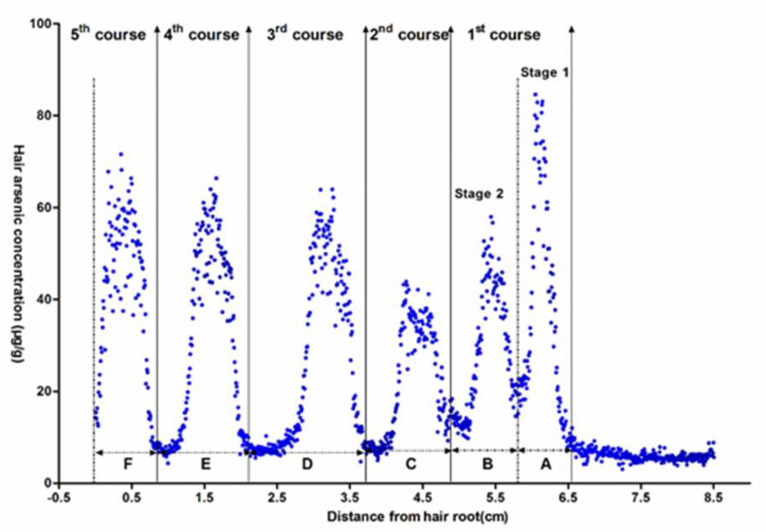
Arsenic concentration profiles for patient No. 8, A-F represents the interval between adjacent sessions.

The hairs obtained from three patients (patient Nos. 6 to 8) were ablated and validated using the same procedure. Taking patient No. 8 as an example, as shown in Figure 3, the As concentration distribution profiles of six successive cycles were separated by five lower-level periods. An unexpected cessation of As_2_O_3_ administration occurred due to liver dysfunction on the 11th day after the beginning of therapy. As a result, the first course was divided into two stages. These results demonstrate that the longitudinal analysis of As along hair can not only reveal the variable uptake of As_2_O_3_ but also represent the intermittent dosing of As_2_O_3_.

The time of As_2_O_3_ administration was estimated according to the mathematical model. The accuracy was calculated by dividing the estimated time by the actual time, which was then normalized to 100%. The deviation was evaluated according to the difference between the estimated time and the actual time.

First, the time between the first day of As_2_O_3_ administration and the sampling day was calculated. The entire hair length between the initial rise and final decrease in As concentration was approximately 5.86 cm, and the estimated time was 142.2 days. According to the medical profile, the patient had received As_2_O_3_ treatment for 150 non-continuous days during the entire therapy period. The accuracy was 94.8%, and the deviation was -7.8 days. Secondly, the intervals between the two adjacent courses of As_2_O_3_ administration (A to F, as shown in Figure 3) were calculated according to the mathematical model. The accuracy ranged from 80.38 to 111.09%, and the deviations varied from −8.83 to 1.89 days, as shown in Table 3. Furthermore, the continuous days of As_2_O_3_ administration in each course were calculated by the mathematical model, which has not been reported in other studies. The hair length between the initial increase and decrease in As concentration in each As peak varied from 0.45 to 0.61 cm, corresponding to 10 to 14 continuous days of As_2_O_3_ administration. The accuracy ranged from 91.93 to 109.20%, and the deviations varied from −1.13 to 0.92 days, as shown in Table 4.

In general, a mathematical model inference of the time of As entry into the body was established using the estimated values of hair length and growth rate corresponding to the axial variation trend of As in hair, accurately inferring the entry time of As.

### 3.5. Application of One Actual Case

**Figure 4:**
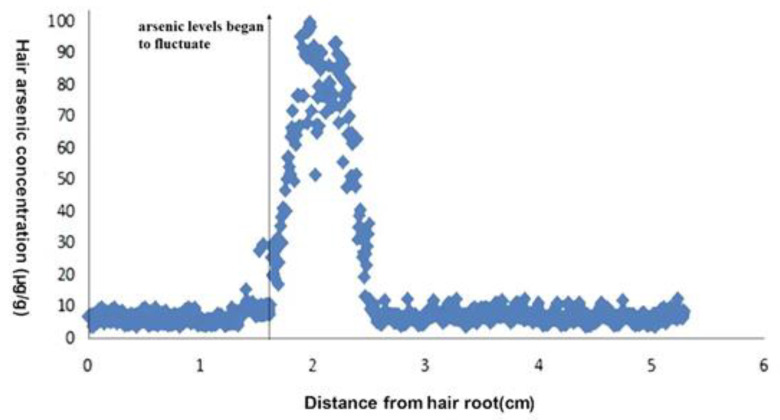
Concentration along hair from a victim of arsenic poisoning.

On 17 April 2019, one victim was hospitalized with abdominal pain, nausea and vomiting after ingesting a small amount of As-containing water. To confirm that these symptoms were caused by As, we first tested the patient’s biometric materials (blood and urine). As concentrations in blood and urine were both in the normal range. We then collected hair from the patient on 30 May for identification (the total length of the hair was about 5 cm). We observed that the As levels began to fluctuate significantly at a distance of 1.632 cm from the root (as shown in Figure 4), and we deduced from the above formula that the patient had been poisoned approximately 39 days prior (i.e., 21 April). In combination with the confession of a suspect, the poisoning time of the victim and the results of the laboratory study proved useful in determining the criminal facts of the case, considerably assisting criminal investigators.

## 4. Discussion

Previous research were likely reported inaccurate exposure information due to poor spatial resolution. However, due to the high spatial resolution, the accuracy of As exposure frequency was inferred in this study. Taking patient No. 8 as an example, the results show that six As concentration peaks occurred, as shown in Figure 3; however, clinical data show that the patient had received As only five times. The detected exposure frequency results contradict the actual course of treatment. We found that the unexpected cessation of As_2_O_3_ administration occurred due to liver dysfunction on the 11th day of therapy. As a result, the first course of treatment was divided into two stages. This example demonstrates that the proposed method accurately determined As exposure frequency. However, it was difficult to distinguish between the two stages in the first course of treatment in previous research, as the hair growth length of the patient only about 0.3 cm during the drug withdrawal time, and it was difficult to accurately cut such a short hair by hand, which led to the loss of As exposure frequency information.

Many authors have shown that As exposure time can be estimated according to the location of As peaks in hair samples [14,15,16]. However, in previous studies, researchers were not able to detect variations in As concentrations along hair in real time and in situ. Due to poor spatial resolution, exposure time error could only be measured in months. In this study, As exposure time was estimated using a high-spatial-resolution method combined with the hair growth rate, and we found that the estimated hair growth rate was similar to values reported in Chinese people [19]. Estimated As exposure time exhibited satisfactory agreement with the actual exposure time. The estimation error was within the range of few days, whereas in previous research the estimation error was in the range of a few months, indicating that the proposed method effective improved estimation accuracy.

The determination of exposure frequency and time is useful for criminal investigation and environment monitoring with respect to compensation for victims and punishment of perpetrators. The results of the present study can be used to prevent the loss of information about As exposure frequency. Furthermore, a strategy for accurately estimating As exposure time was proposed, which can be applied to estimate the time of exposure to other toxic metals. Accurate exposure time estimation can help to narrow the time range of criminal investigations in intentional poisoning cases and environment exposure monitoring.

## 5. Study Limitations

Compared with previously published research, although confirmed the frequency and time of arsenic exposure, the present study is subject to limitations with respect to deviations between the time estimate and the actual time of exposure. In future studies, we will aim to optimize the experimental conditions to the greatest extent possible to improve the accuracy of the results.

## 6. Conclusions

In this study, occipital hairs of APL patients treated with As_2_O_3_ were used as research objects, and LA-ICP-MS technology was used to analyze the arsenic elemental contents in a single hair in real time and in situ to obtain information about the distribution of arsenic. As exposure frequency was estimated according to the characteristics of arsenic distribution in hair based on the number of As concentration peaks, and the As exposure time was estimated using according to variations in As concentrations, combined with the estimated hair growth rate. This study provides a new method of estimating the frequency and timing of toxic elemental exposure in the body, and the reported results could be useful for future forensic science studies, as well as archaeological and environment monitoring applications.

## Figures and Tables

**Table 1:** Detailed medical profiles of all patients.

Patient No.	1st Course		2nd Course		3rd Course		4th Course		5th Course		6th–10th Course	
	AT (Day)	WT (Day)	AT (Day)	WT (Day)	AT (Day)	WT (Day)	AT (Day)	WT (Day)	AT (Day)	WT (Day)	AT (Day)	WT (Day)
1	22	18	14	16	14	15	14	18	14	-	-	-
2	14	15	14	45	14	18	14	-	-	-	-	-
3	14	58	14	17	14	58	14	-	-	-	-	-
4	14	57	14	18	14	19	-	-	-	-	-	-
5 ^a^	14	18	14	15	14	18	14	67	14	-	14	-
6	14	17	14	-	-	-	-	-	-	-	-	-
7	14	21	14	21	14	51	14	17	14	-	-	-
8 ^b^	10	7	14	17	14	31	14	17	14	-	-	-
	11	15									-	-

AT, administration time; WT, withdraw time. ^a^ Patient No. 5 received a 10-course As_2_O_3_ treatment comprising an oral dose of 10 mg As_2_O_3_ for 14 continuous days for each course; the interval for the 6th to 10th courses ranged from 13 days to 50 days. ^b^ Cessation of As_2_O_3_ administration occurred during the first course for patient No. 8; therefore, the first course was divided into two stages.

**Table 2:** Hair growth rate measured by arsenic longitudinal distribution along hair strands.

Patient No.	Sex (M/F)	Age (y)	Hair Growth Rate: Mean ± SD (Range) (μm/day)		Intraindividual CV (%) (*n* = 3)	Interindividual CV (%) (*n* = 15)
1	F	25	439 ± 15 (406,471)		3.39	6.37
2	F	28	424 ± 23 (370,477)		5.48	2.82
3	M	35	403 ± 24 (348,459)		5.94	−2.18
4	F	30	418 ± 29 (343,493)		6.99	1.45
5	F	46	398 ± 12 (374,423)		2.94	−3.36
			Mean	412 ± 8	1.93	1.02

M, male; F, female; y, years; SD, standard deviation; CV, coefficient of variation.

**Table 3:** Estimation results for patient No. 8.

Hair Length (cm)		Actual Time (day)	Estimated Time (day)	Accuracy (%)	Time of Deviation (day)
A	0.78	17	18.89	111.09	1.89
B	1.04	26	25.24	97.08	−0.76
C	1.35	31	32.77	105.71	1.77
D	1.49	45	36.17	80.38	−8.83
E	1.20	31	29.13	93.97	−1.87
F	0.71	20	17.24	86.20	−2.76
A→F	6.57	170	159.89	89.04	−10.53

**Table 4:** Estimation results of continuous days of As_2_O_3_ administration in each course for patient No. 8.

Course No.		Hair Length (cm)	Actual Time (Day)	Estimated Time (Day)	Accuracy (%)	Time of Deviation (Day)
1	Stage 1	0.45	10	10.92	109.20	0.92
	Stage 2	0.46	11	11.17	101.55	0.17
2		0.60	14	14.56	104.00	0.56
3		0.53	14	12.87	91.93	−1.13
4		0.55	14	13.35	95.36	−0.65
5		0.61	14	14.81	105.79	0.81

## Data Availability

The data presented in this study are available on request from the corresponding authors.
